# Identification of novel biomarkers affecting the metastasis of colorectal cancer through bioinformatics analysis and validation through qRT-PCR

**DOI:** 10.1186/s12935-020-01180-4

**Published:** 2020-03-30

**Authors:** Wenping Lian, Huifang Jin, Jingjing Cao, Xinyu Zhang, Tao Zhu, Shuai Zhao, Sujun Wu, Kailu Zou, Xinyun Zhang, Mingliang Zhang, Xiaoyong Zheng, Mengle Peng

**Affiliations:** 1 Department of Clinical Laboratory, Henan No. 3 Provincial People’s Hospital, Zhengzhou, 450006 Henan China; 2 Department of Blood Transfusion, The First Affiliated Hospital of Zhengzhou University, Zhengzhou, 450052 Henan China; 3 Department of Basis Medicine, Henan Medical College, Zhengzhou, 451191 Henan China; 4 Department of Medical Affair, Henan No. 3 Provincial People’s Hospital, Zhengzhou, 450006 Henan China; 5 Department of Clinical Laboratory, Zhecheng People’s Hospital, Shangqiu, 476000 Henan China; 6 Medical College of Zhengzhou University, Zhengzhou, 450052 Henan China; 7 Department of Anorectal Surgery, Henan No. 3 Provincial People’s Hospital, Zhengzhou, 450006 Henan China; 8 Henan Province Engineering Laboratory for Clinical Evaluation Technology of Chinese Medicine, The First Affiliated Hospital of Henan University of Traditional Chinese Medicine, Zhengzhou, 450000 China; 9 Department of Digestion, Henan No. 3 Provincial People’s Hospital, Zhengzhou, 450006 Henan China

**Keywords:** Colorectal cancer, Metastasis, Prognosis, Biomarker, WGCNA

## Abstract

**Background:**

Tumor progression and distant metastasis are the main causes of deaths in colorectal cancer (CRC) patients, and the molecular mechanisms in CRC metastasis have not been completely discovered.

**Methods:**

We identified differentially expressed genes (DEGs) and lncRNAs (DELs) of CRC from The Cancer Genome Atlas (TCGA) database. Then we conducted the weighted gene co-expression network analysis (WGCNA) to investigate co-expression modules related with CRC metastasis. Gene ontology (GO), Kyoto Encyclopedia of Genes and Genomes (KEGG) pathway analysis, DEG-DEL co-expression network and survival analyses of significant modules were also conducted. Finally, the expressions of selected biomarkers were validated in cell lines by quantitative real-time PCR (qRT-PCR).

**Results:**

2032 DEGs and 487 DELs were involved the construction of WGCNA network, and greenyellow, turquoise and brown module were identified to have more significant correlation with CRC metastasis. GO and KEGG pathway analysis of these three modules have proven that the functions of DEGs were closely involved in many important processes in cancer pathogenesis. Through the DEG-DEL co-expression network, 12 DEGs and 2 DELs were considered as hub nodes. Besides, survival analysis showed that 30 DEGs were associated with the overall survival of CRC. Then 10 candidate biomarkers were chosen for validation and the expression of CA2, CHP2, SULT1B1, MOGAT2 and C1orf115 were significantly decreased in CRC cell lines when compared to normal human colonic epithelial cells, which were consistent with the results of differential expression analysis. Especially, low expression of SULT1B1, MOGAT2 and C1orf115 were closely correlated with poorer survival of CRC.

**Conclusion:**

This study identified 5 genes as new biomarkers affecting the metastasis of CRC. Besides, SULT1B1, MOGAT2 and C1orf115 might be implicated in the prognosis of CRC patients.

## Background

Colorectal cancer (CRC) is the third most common cancer and the second leading cause of cancer-related deaths in broad areas of the world, accounting for nearly 1.2 million new patients and 600,000 deaths/year [1, 2]. It is reported that about 50–60% of patients with CRC die from distant metastasis [3]. Although multiple treatments, such as surgery, chemotherapy, radiotherapy, targeted therapy, and immunotherapy, have shown to reduce the relapse and improve the survival of CRC patients, the 5-year survival rate of this malignancy is still poor [4, 5]. Therefore, it is vital to uncover the critical new biomarkers and the underlying mechanisms associated with CRC metastasis.

So far, a variety of recent cancer profiling studies have focused on RNA-sequencing (RNA-Seq), a rapidly maturing development of the next-generation sequencing technology. As one prominent example of the renowned public databases, the Cancer Genome Atlas (TCGA) provides a platform of RNA-Seq with mRNA, miRNA and lncRNA data of various cancers. Long noncoding RNAs (lncRNAs) are defined as a class of transcripts with a length of more than 200 nucleotides, with limited potential of protein-coding capacity [6]. LncRNAs have mechanistically diverse functions in the cell, and in the nucleus have been shown to regulate gene expression either in cis or in trans by recruiting chromatin-modifying complexes to promoters of target genes [7, 8]. Weighted gene co-expression network analysis (WGCNA) has been established as an effective data mining method for finding clusters or modules of highly correlated biomolecules and identifying intra modular “hubs”, including genes [9] miRNAs [10] and lncRNAs [11]. More importantly, WGCNA analyzes the relationships between modules and sample traits, which provides an effective way to explore the mechanisms behind certain traits [12]. In 2018, Zhou et al. [13] had conducted a WGCNA to predict pathological stage-related miRNA and gene modules of colon cancer. However, to the best of our knowledge, studies associated with gene and lncRNA modules of CRC metastasis have not been reported.

In this study, we successfully identified a set of differentially expression genes (DEGs) and lncRNAs (DELs) by integratively analyze RNA-Seq data of colon adenocarcinoma (COAD) and rectal adenocarcinoma (READ) patients from TCGA database. Then based on the merged DEGs and DELs of COAD and READ, we conducted WGCNA and module-trait relationships to investigate significant modules related with CRC metastasis. Subsequently, Gene Ontology (GO) and Kyoto Encyclopedia of Genes and Genomes (KEGG) pathway enrichment of significant modules were conducted. In addition, DEG-DEL co-expression network and survival analysis of the top 100 nodes of significant modules were conducted to find important biomarkers. Finally, we chose 10 candidate biomarkers for validation in CRC cell lines and normal human colonic epithelial cells by quantitative real-time PCR (qRT-PCR). Thus, this comprehensive analysis might provide a meaningful contribution to exploring potential biomarkers or therapeutic targets of CRC metastasis at the transcriptomic level.

## Materials and methods

### Study design and data processing

**Fig. 1:**
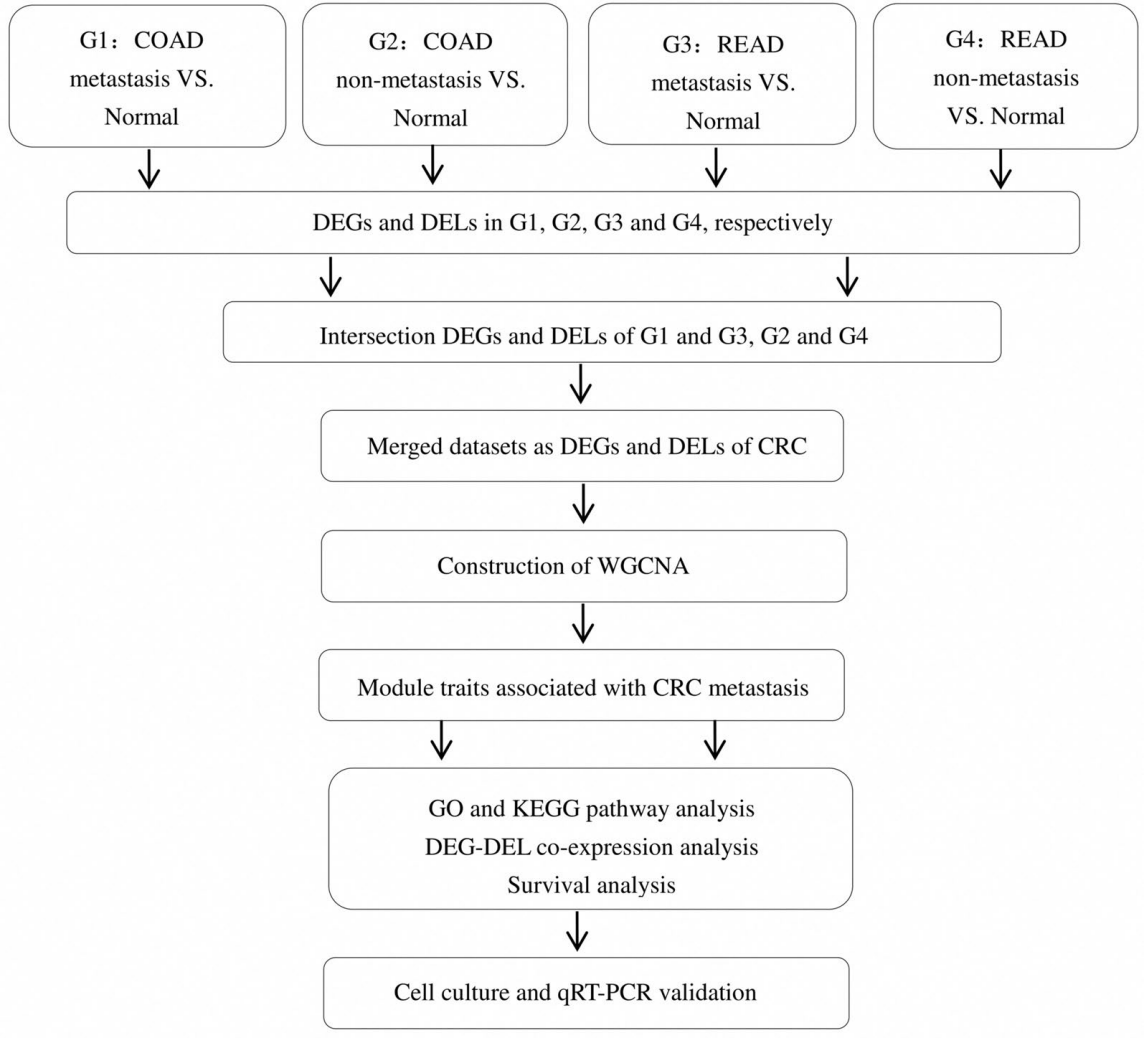
Flow chart of data processing, analysis and validation

The study design was showed in a flow diagram (Fig. 1), including TCGA-based RNA-seq data aggregation, multiple bioinformatics analysis and validation.

The RNA-seq data and the corresponding patient clinical data of COAD and READ patients was obtained from the TCGA database, which were derived from IlluminaHiSeq RNA-Seq platform. Patients with other tumors or patients without clinical information of metastasis were excluded. The mRNA and lncRNA expression data included a total of 401 COAD samples consisting of 336 metastasis and 65 non-metastasis samples, and a total of 154 READ samples consisting of 130 metastasis and 24 non-metastasis samples. No ethical issues were involved, because the sequencing data were obtained by using TCGA database.

### Identification of DEGs and DELs

The limma package of R was used to screen the DEGs and DELs between COAD and READ metastasis, non-metastasis and normal samples, setting the false discovery rate (FDR) < 0.05, fold change (FC) > 1.5 or FC < 0.5 and P < 0.05 as the cut-off criterion. These samples were divided into four groups: G1 (COAD metastasis vs. normal); G2 (COAD non-metastasis vs. normal); G3 (READ metastasis vs. normal); G4 (READ non-metastasis vs. normal). According to the TCGA project’s large-scale study of CRC specimens, the pattern of genomic alterations in CRC tissue is the same regardless of whether tumor origin is in the colon or the rectum, leading to the conclusion that these two cancer types can be grouped as one [14]. Thus, we obtained the merged datasets of COAD and READ as DEGs and DELs of CRC for WGCNA analysis.

### Construction of WGCNA

In this study, the merged DEGs and DELs of CRC were applied to construct co-expression modules using “WGCNA” package in R [12]. The Pearson’s correlation matrices cor (i, j) which calculated the connection strength between each pair of retained DEGs and DELs from the corresponding expression levels was performed. Then a weighted adjacency matrix was computed as follows:

$${\text{a}}_{\text{ij}} = \left( {0.5 \times \left( {1 + {\text{cor}}\left( {{\text{i}},{\text{ j}}} \right)} \right)} \right)^{\upbeta} ,$$

where a_ij_ represented the Pearson’s correlation coefficient between nodes i and j. β was a soft-thresholding parameter that could emphasize strong correlations between nodes and penalize weak correlations [15]. Next, the topological overlap measure (TOM) representing the overlap in shared neighbors, was derived from the adjacency matrix, and the value (1 − TOM) was designated to the distance for identification of hierarchical clustering nodes and modules [16]. Clusters were obtained from the dendrogram by applying the dynamic tree cutting technique.

### Identification of significant modules associated with CRC metastasis

The WGCNA algorithm uses module eigengenes (MEs) which were considered as the major component in the principal component analysis for each gene module to assess the potential correlation of gene modules with clinical traits. Information on the clinical characteristics (metastasis and non-metastasis) were selected as traits to identify significant co-expression module related with CRC metastasis. Module-trait relationships were calculated according to the correlation between modules and traits by Pearson’s correlation tests, and when P < 0.05 was defined to be significantly correlated. DEGs and DELs in significant modules were then exported for further analysis.

### Functional enrichment analysis of significant modules

In significant modules, the Database for Annotation, Visualization and Integrated Discovery (DAVID) was used to conduct GO and KEGG pathway analysis of DEGs. Enriched GO terms and KEGG pathways were identified with the cut-off criterion of P < 0.05.

### Construction of DEG-DEL co-expression networks

To investigate the relationship between DEGs and DELs, we constructed the DEG-DEL co-expression networks based on the top 100 nodes of significant modules. Significant correlation pairs were used to construct the network based on Pearson correlation coefficients. Finally, the differential co-expression network graphs were visualized and analyzed using Cytoscape software (Version 3.5.1).

### Survival analysis

Survival analysis of the top 100 DEGs and DELs in each significant modules were performed by using the Gene Expression Profiling Interactive Analysis (GEPIA) database [17]. The Kaplan–Meier curves were plotted in the GEPIA database including 362 CRC patients. And according to the median risk score, CRC patients were divided into high- and low-risk groups. P < 0.05 was considered statistically significant.

### Cell culture

CRC cell lines HCT**-**8, SW1116 and DLD-1 and normal human colonic epithelia cell line (NCM460) were purchased from The Shanghai Institutes for Biological Sciences of the Chinese Academy of Sciences (Shanghai, China). All the cells were inoculated to RPMI-1640 culture solution complementary with 10% fetal bovine serum (ThermoScientific HyClone, Logan, UT, USA), and maintained at 37 °C in humidified atmosphere containing 5% CO_2_, with liquid exchanging and passaging every 3–4 days.

### RNA extraction and qRT-PCR analysis of candidate biomarkers

HCT-8, SW1116, DLD-1and NCM460 cells RNA were extracted using R Neasy Purification Kit (Qiagen) (Cat. #74101, Qiagen, Germany) according to the manufacturer’s instruction. RNA yield and purity were determined by measuring the absorbance (Abs) at 260 and 280 nm. Only RNA samples with Abs260 nm/Abs280 nm ratio > 1.8 were used. After that, the cDNA was synthesized using 1 μg of total RNA by ReverTra Ace qPCR RT Kit (Toyobo, China) and qRT-PCR was performed using 400 ng cDNA per 25 μl reaction. Gene-specific primers were designed using online primer designing tools primer-blast. The primer sequences are listed in Table 1.

**Table 1:** The primers for qRT-PCR

Target	Sequence (5′–3′)
CA2 (F)	TCGTGGCCTCCTTCCTGAAT
CA2 (R)	CTGCTGACGCTGATGGGTTC
CHP2 (F)	TACCTGAGCCGCATGGATCTCC
CHP2 (R)	GAGCCAAGACCCTGACAAAGCC
SULT1B1 (F)	CTCGTAATGCCAAGGATGTTTCA
SULT1B1 (R)	CTTGATTTCCTCCTTTGGATTCTCT
SLC51B (F)	CAGGAGCTGCTGGAAGAGATGC
SLC51B (R)	CTGCCAGGGCAAGGATGGAATG
SMPDL3A (F)	GCAGTAGCAAACCTCTGGAAAC
SMPDL3A (R)	CTGGGTCAGTCTTGTTCAGTGTC
MOGAT2 (F)	GGTATCTGGACCGAGACAAGCC
MOGAT2 (R)	GTGGAAGCCCGCAATGTAGTT
ITM2C (F)	CGAGATAACTTCTTCCGCTGTG
ITM2C (R)	CACAATGGTGGTGTTGAGTTCG
LRRC19 (F)	TATGGAAACCTATGGAACTGCTCT
LRRC19 (R)	GCTGATGGGCTGAAAATGAATA
C1orf115 (F)	AGTACGGCAAGAATGTCGGGA
C1orf115 (R)	AGGATACCACGCTGGTGGCTA
RP11-396O20.2 (F)	GAATGCTGGCAAAGTTCGTGA
RP11-396O20.2 (R)	TCTCCTTGGGATGATGGTGC

### Statistical analyses

Statistical analyses were performed with R software 3.4.0 and SPSS 19.0. Two-tailed Student’s t-test was used for significance of differences between two groups. P < 0.05 was considered to be significant.

## Results

### Identification of DEGs and DELs

**Fig. 2:**
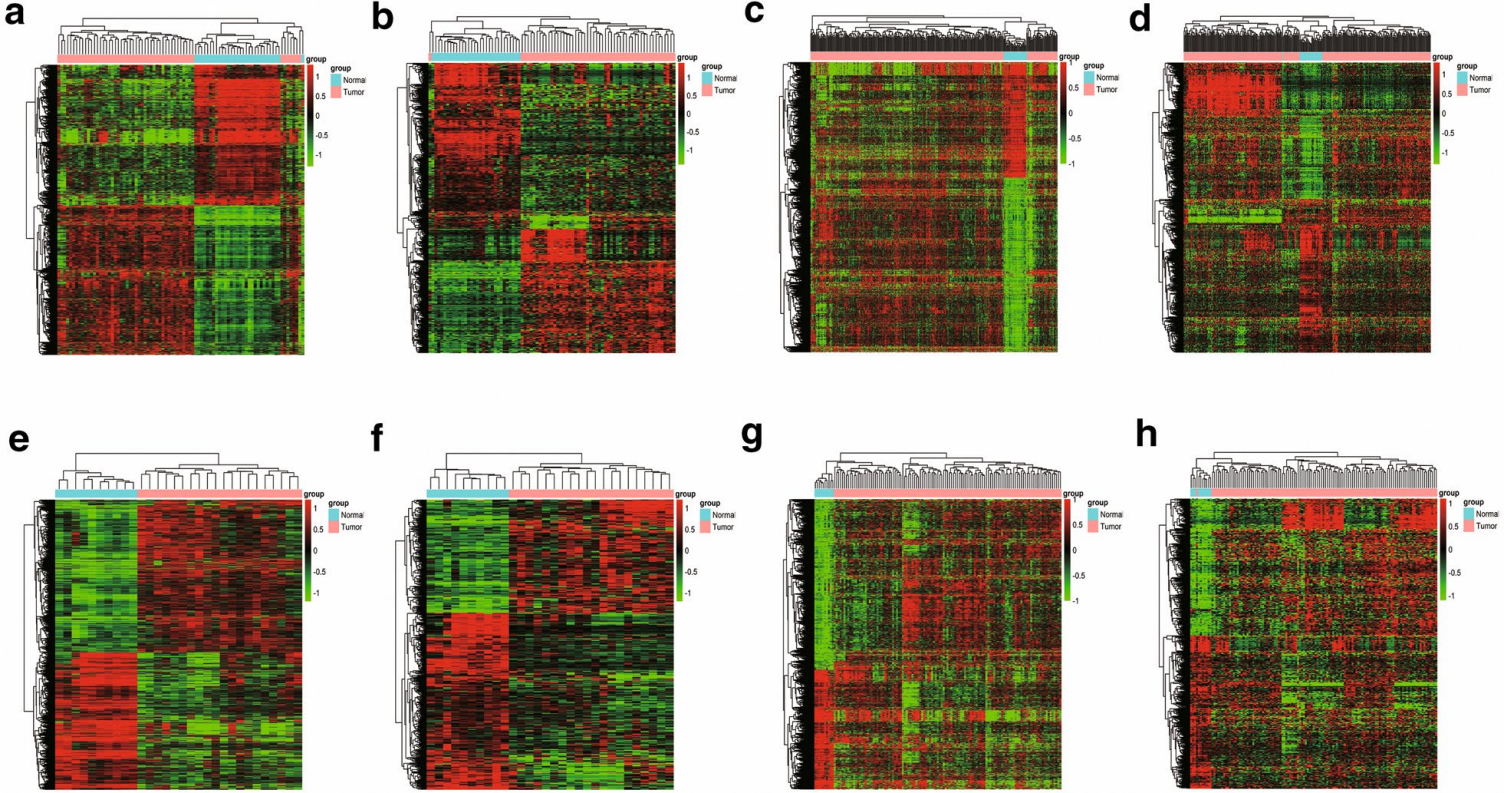
Heatmap for hierarchical cluster analysis of DEG and DEL expression levels change between **a**, **b** COAD metastasis and normal tissues (G1); **c**, **d** COAD non-metastasis and normal tissues (G2); **e**, **f** READ metastasis and normal tissues (G3); **g**, **h** READ non-metastasis and normal tissues (G4). The red and green colors represent higher expression levels and lower expression levels of DEGs and DELs, respectively

The heatmap of DEGs and DELs of these four groups were shown in Fig. 2. As these two cancer types can be grouped as one [14], we finally merged the intersecting datasets of COAD and READ as a whole of CRC. Before that, the intersection datasets of COAD and READ were obtained by selecting the intersection of G1 and G3, G2 and G4, respectively (Fig. 1). Thus, a total of 2032 DEGs including 1101 (54.2%) up-regulated and 931 (45.8%) down-regulated genes, and 487 DELs including 214 (43.9%) up-regulated and 273 (56.1%) down-regulated lncRNAs were obtained. Subsequently, we performed the following analysis based on this part of data.

### Construction of WGCNA network

**Fig. 3:**
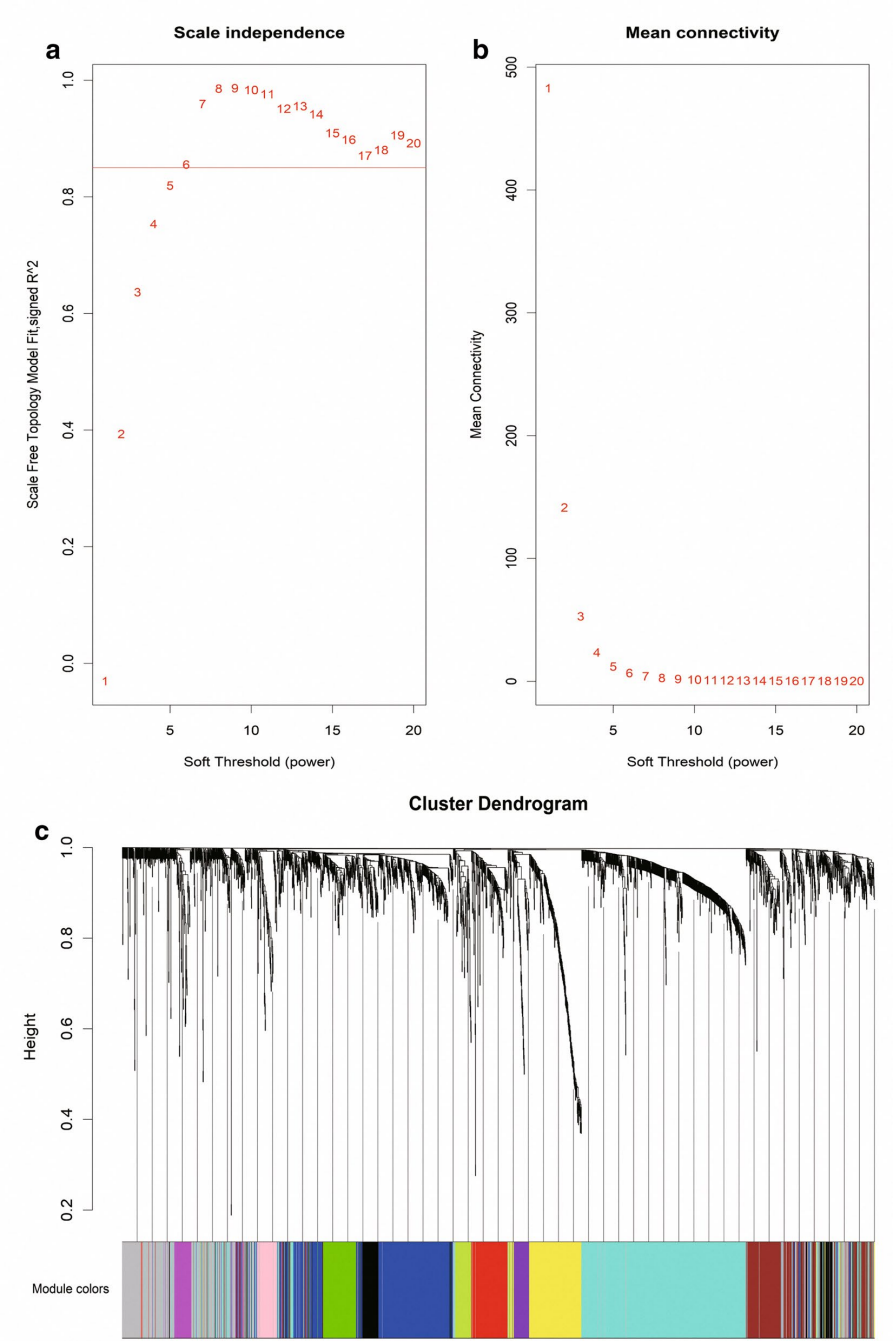
**a** Scale independence and **b** mean connectivity analysis for various soft-thresholding powers. **c** Clustering dendrogram of DEGs and DELs based on a dissimilarity measure (1 − TOM), with dissimilarity based on topological overlap. Each color below represents one co-expression module (gray represents unassigned DEGs and DELs)

Altogether, 2032 DEGs and 487 DELs were involved the construction of WGCNA network. Then the soft threshold was determined by scale independence and mean connectivity analysis of modules with different power values ranging from 1 to 20. In this study, when the power value (β) was set to 6, the scale independence value achieved 0.8 and lower mean connectivity (Fig. 3a, b). Thus, β = 6 was selected to produce a hierarchical clustering tree with different colors representing different modules. After putting all these DEGs and DELs with similar expression patterns into modules by average linkage clustering (Fig. 3c), a total 12 modules (MEpurple, MEred, MEgreenyellow, MEyellow, MEturquoise, MEgreen, MEpink, MEblue, MEblack, MEbrown, MEmagenta and MEgrey) with different DEGs and DELs were identified and displayed with different colors, the distribution of DEGs and DELs among these modules were summarized in Table 2.

**Table 2:** The number of DEGs and DELs in the 12 modules

Module color	All number	DEGs	DELs
PURPLE	58	57	1
RED	123	91	32
Greenyellow	53	45	8
Yellow	200	167	33
Turquoise	659	570	89
Green	124	101	23
Pink	66	3	63
Blue	399	374	25
Black	121	114	7
Brown	250	222	28
Magenta	66	19	47
Grey	400	269	131

### Identification of significant modules associated with CRC metastasis

**Fig. 4:**
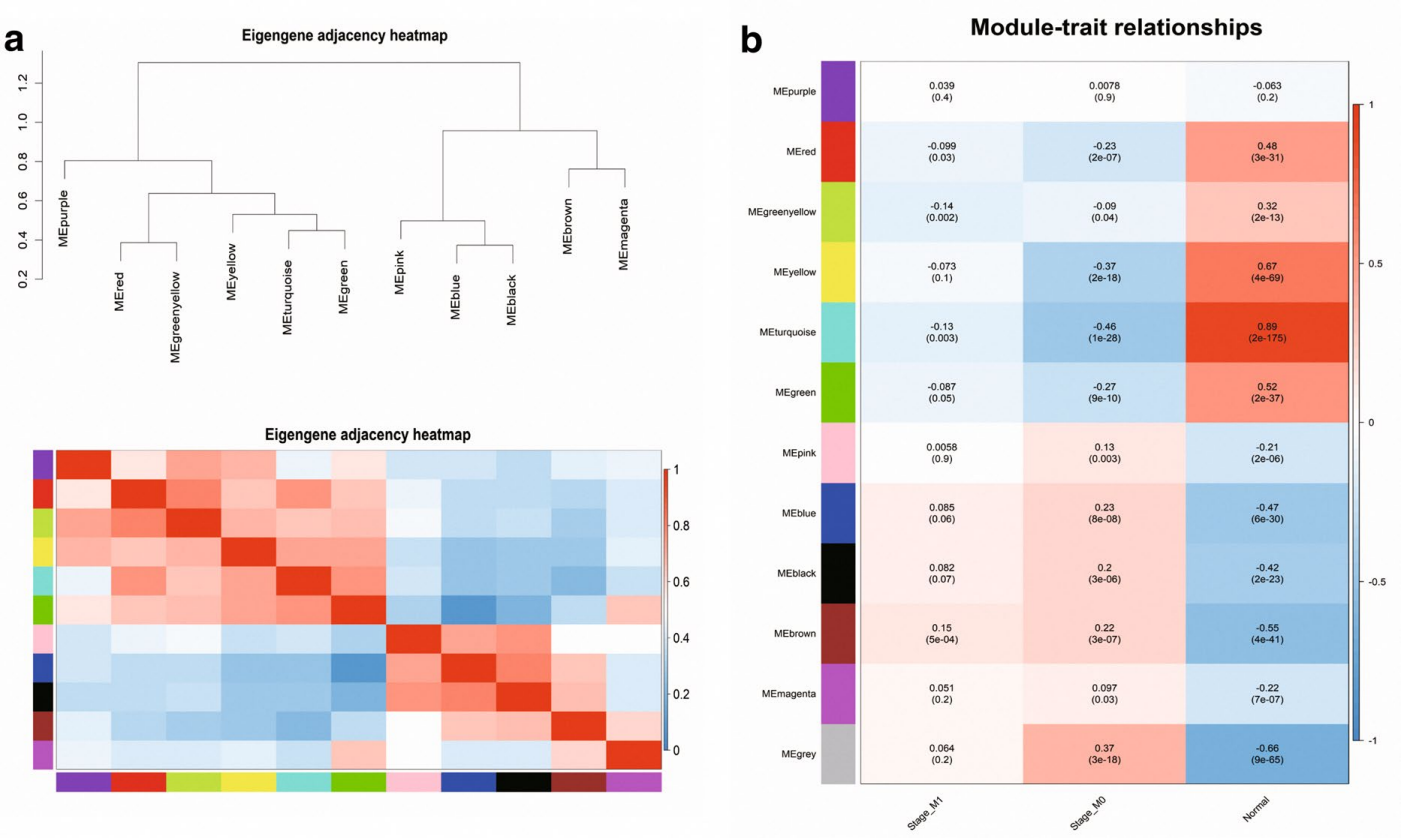
**a** Hierarchical clustering of module and heatmap plot of the eigengene adjacencies. **b** Module-trait relationships. Each row corresponds to a module eigengene, column to a trait. The correlation coefficient (upper number) and corresponding P-value (lower number) in each cell resulted in the correlation between the gene module and the clinical trait. M0: cancer, non-metastasis; M1: cancer, metastasis

The eigengene adjacency network and hierarchical clustering dendrogram of the eigengenes were shown in Fig. 4a, b. Four modules (red, greenyellow, turquoise and brown) associated with CRC metastasis were identified by WGCNA analysis with P < 0.05. There were 91 DEGs and 32 DELs in a red module, 45 DEGs and 8 DELs in a greenyellow module, 570 DEGs and 89 DELs in a turquoise module, 222 DEGs and 28 DELs in a brown module (Table 1). Among them, the brown module was a positive module, the red, greenyellow and turquoise module were negative modules correlated with CRC metastasis (Fig. 4b). According to their P value, we chose the greenyellow, turquoise and brown module which had more significant correlation with CRC metastasis for further analysis.

### Functional enrichment analysis of greenyellow, turquoise and brown modules

**Fig. 5:**
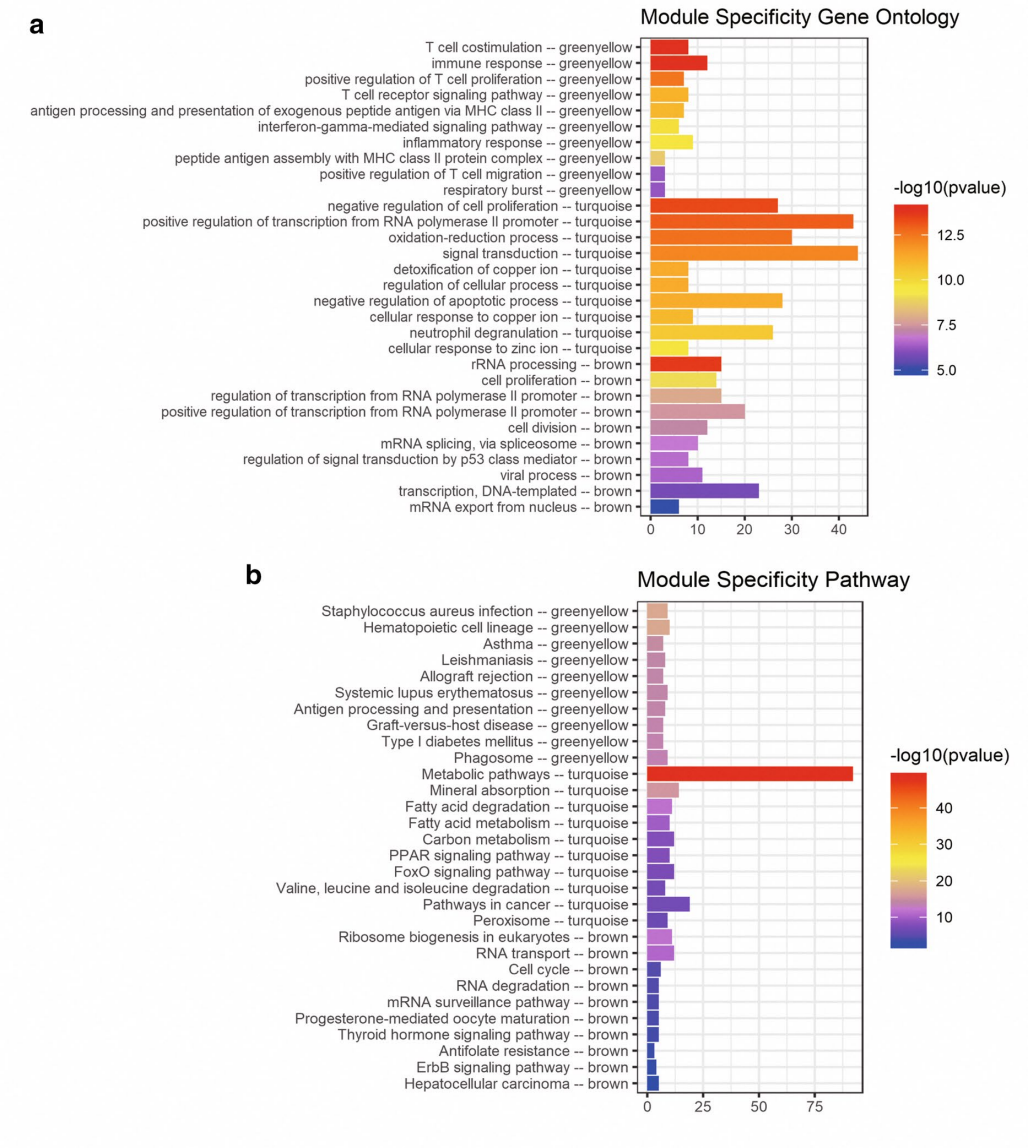
GO (**a**) and KEGG pathway analysis (**b**) of DEGs in greenyellow, turquoise and brown modules

DEGs in the greenyellow, turquoise and brown module were used for GO analysis and KEGG pathway enrichment analysis so as to explore the underlying biological process correlated to CRC metastasis. We could find that DEGs in greenyellow module significantly enriched in T cell costimulation, immune response and positive regulation of T cell proliferation. In the turquoise module, the DEGs were mainly enriched in negative regulation of cell proliferation, positive regulation of transcription from RNA polymerase II promoter and oxidation–reduction process. For the brown module, the DEGs were mainly involved in rRNA processing, cell proliferation and regulation of transcription from RNA polymerase II promoter (Fig. 5a).

The results of KEGG pathway analysis showed that the DEGs were mainly related to staphylococcus aureus infection, hematopoietic cell lineage and asthma in greenyellow module; metabolic pathways, mineral absorption and fatty acid degradation in turquoise module; ribosome biogenesis in eukaryotes, RNA transport and cell cycle in the brown module (Fig. 5b).

### Construction of the DEG-DEL co-expression network

**Fig. 6:**
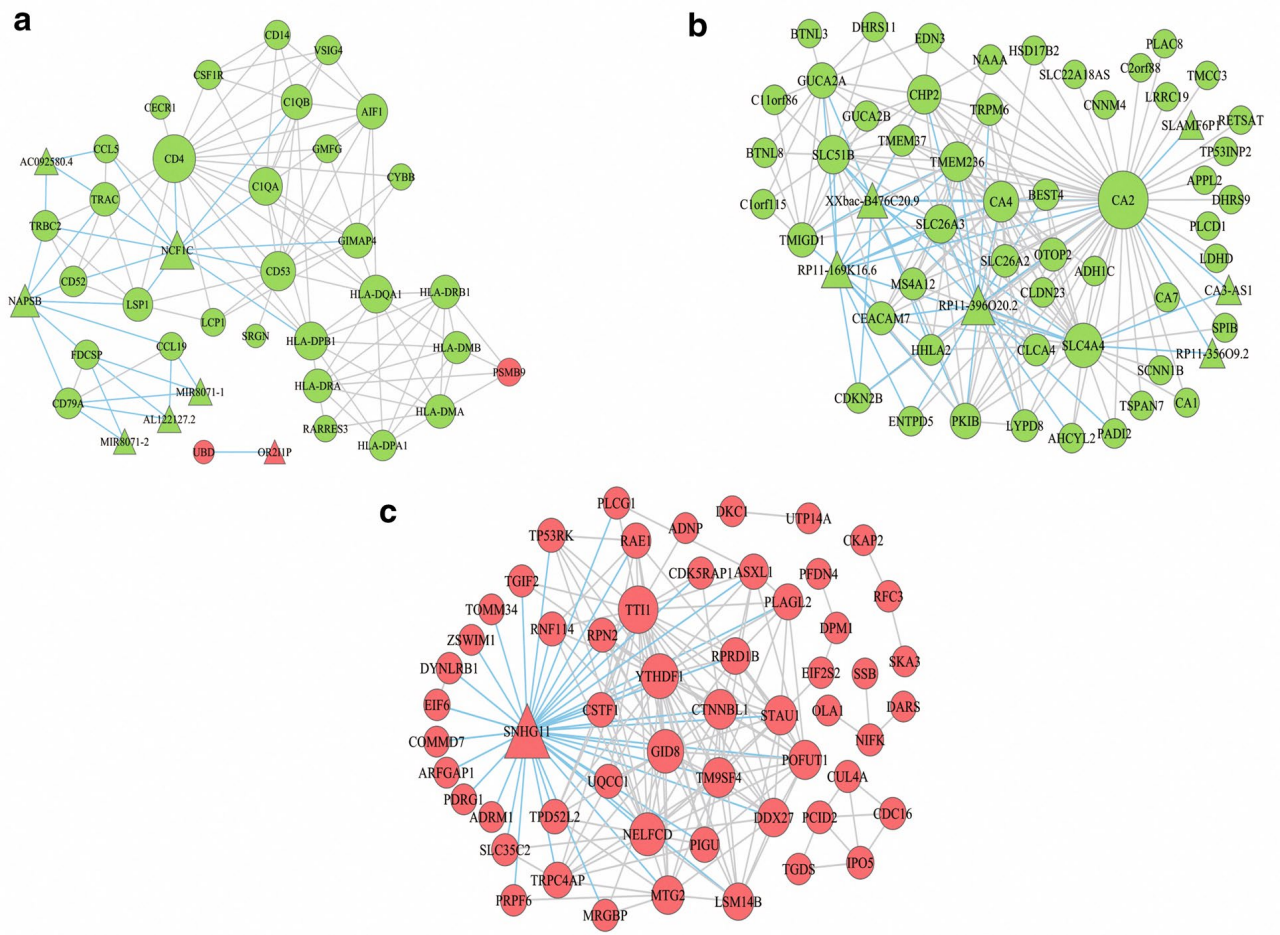
Co-expression pattern of DEGs-DELs in greenyellow, turquoise and brown modules. The circular nodes indicate the DEGs, triangle nodes indicate DELs. Red represents upregulation, while green represents upregulation

DEG-DEL co-expression pattern were constructed based on the correlation analysis between the top 100 nodes (DEGs and DELs) of the turquoise, brown module and all of them in greenyellow module. A higher degree for one node meant that the node played a more important role in this network. In this study, nodes with degree more than 15 were considered as hub nodes. Among the three modules, there were 14 hub nodes were identified, including 12 DEGs (CA2, SLC4A4, CA4, TMEM236, CHP2, SLC26A3, SLC51B, TTI1, YTHDF1, GID8, NELFCD and CD4) and 2 DELs (RP11-396O20.2 and SNHG11) (Fig. 6) (Table 3).

**Table 3:** Nodes with more than 15 connections in the DEG-DEL co-expression network

Node	Degree	Biotype	Module
CA2	51	mRNA	Turquoise
SLC4A4	28	mRNA	Turquoise
CA4	22	mRNA	Turquoise
TMEM236	20	mRNA	Turquoise
RP11-396O20.2	19	lncRNA	Turquoise
CHP2	19	mRNA	Turquoise
SLC26A3	18	mRNA	Turquoise
SLC51B	18	mRNA	Turquoise
SNHG11	37	lncRNA	Brown
TTI1	27	mRNA	Brown
YTHDF1	23	mRNA	Brown
GID8	21	mRNA	Brown
NELFCD	20	mRNA	Brown
CD4	18	mRNA	Greenyellow

### Survival analysis

We performed Kaplan–Meier survival curves of the above-described DEGs and DELs of the three modules to examine whether they were associated with the outcome of CRC patients.We found that 30 DEGs were associated with the survival prognoses of CRC patients (P < 0.05). Especially, SULT1B1, CPT2, LRRC19, SLC26A3, ABCE1, C4orf19, AURKA, GPD1L and NR3C2 were the signiicant prognostic DEGs with P < 0.01. Unfortunately, no DELs were found to be associated with survival of CRC. More information about these survival-associated DEGs is shown in Additional file 1: Figure S1.

### Validation of candidate biomarkers by qRT-PCR

In order to identify new potential biomarkers or therapeutic targets which may play more significant roles in CRC metastasis, we combined hub nodes (12 DEGs and 2 DELs) as well as survival-associated DEGs first. After we excluded some DEGs and DELs which have been reported in CRC, we finally chose the top 10 (CA2, CHP2, SLC51B, ITM2C, LRRC19, SULT1B1, SMPDL3A, MOGAT2, C1orf115 and RP11-396O20.2) as our candidate biomarkers for qRT-PCR validation in cell lines according to their differential expression levels.

**Fig. 7:**
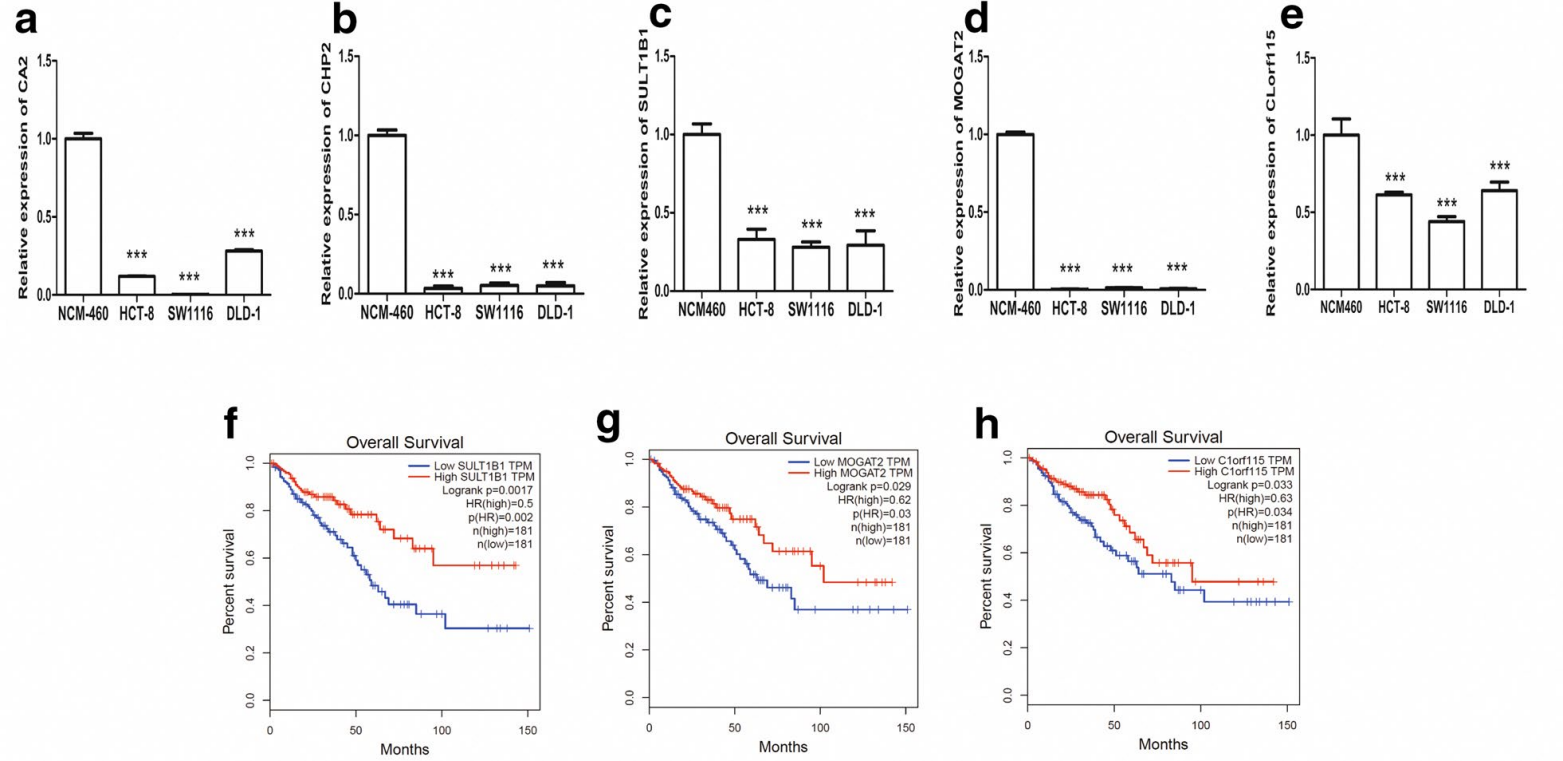
**a**–**e** qRT-PCR validation of the expression of CA2, CHP2, SULT1B1, MOGAT2 and C1orf115 in cell lines. **f**–**h** Survival analysis of SULT1B1, MOGAT2 and C1orf115. The patients were stratified into high-level group and low-level group according to median expression. ***P < 0.001

As shown in Fig. 7, the expression levels of CA2, CHP2, SULT1B1, MOGAT2 and C1orf115 were significantly decreased in CRC cell lines compared with NCM460 (P < 0.001), which were consistent with the results of differential expression analysis, suggesting that the results were convincing (Fig. 7a–e). Moreover, low expression of SULT1B1, MOGAT2 and C1orf115 were closely correlated with poorer survival of CRC (Fig. 7f–h).

## Discussion

Tumor progression and distant metastasis are the main causes of deaths in CRC patients, and the processes of which are complicated that involve a series of complex genetic and epigenetic changes [18, 19]. Therefore, it is of great urgency to detect new molecules and the underlying mechanisms associated with CRC metastasis so as to provide potential biomarkers or therapeutic targets for CRC.

In this study, a total of 12 co-expression modules were constructed by WGCNA based on 2032 DEGs and 487 DELs to investigate metastasis-associated modules of CRC. The results showed that four modules were significantly associated with CRC metastasis. We finally selected greenyellow, turquoise and brown module which had more significant correlation with CRC metastasis for further analysis. DEGs in these three modules were majorly enriched in immune response, positive regulation of transcription from RNA polymerase II promoter, cell proliferation, rRNA processing, etc. In addition, these DEGs were also involved in metabolic pathways, pathways in cancer, etc.

The co-expression networks of the three significant modules provided an insight of correlation between DEGs and DELs. Then 12 DEGs (CA2, SLC4A4, CA4, TMEM236, CHP2, SLC26A3, SLC51B, TTI1, YTHDF1, GID8, NELFCD and CD4) and 2 DELs (RP11-396O20.2 and SNHG11) which had higher connections with other nodes were considered as hub nodes. This means that these biomarkers might play an important role in the metastasis mechanism of CRC. After that, we conducted survival analysis of the above-described nodes to determine valuable predictive factors for CRC patient’s survival. Then we illustrated that 30 DEGs were significantly associated with the overall survival time. Moreover, based on the hub nodes and survival-associated DEGs, we selected 9 genes (CA2, CHP2, SLC51B, ITM2C, LRRC19, SULT1B1, SMPDL3A, MOGAT2 and C1orf115) and 1 lncRNA (RP11-396O20.2) for validation. Finally, CA2, CHP2, SULT1B1, MOGAT2 and C1orf115 were successfully validated with their low expression in CRC cell lines, while the only lncRNA (RP11-396O20.2) we chose failed to be verified. Among them, the low expression of SULT1B1, MOGAT2 and C1orf115 were closely correlated with poorer survival of CRC.

Carbonic anhydrase 2 (CA2) encodes one of isozymes of carbonic anhydrase which catalyzes reversible hydration of carbon dioxide and plays a pivotal role in tissue pH homeostasis [20]. The expression of CA2 remains controversial in different cancers, it was reported to be upregulated in urinary bladder cancers [21], and CA2 autoantibody titers in gastric cancer patients were found higher compared to healthy subjects [22], while in esophageal adenocarcinoma, its expression was downregulated [23]. A previous study [24] conducted integrated bioinformatics analysis has found that lower expression of CA2 had a shorter overall survival compared to those with higher expression in COAD patients, its RNA and protein expression level were also validated in TCGA and the Human Protein Atlas, but without any experimental validation. And their study did not involve the data of READ as well as the association with metastasis of CRC. For our study, we successfully verified that low expression of CA2 might play roles in the metastasis mechanism of CRC. calcineurin homologous protein isoform 2 (CHP2) is expressed in normal intestinal epithelia and also epithelium-like cell line [25], and it might act as a potential role in transmembrane Nat/Ht exchange which can protect cells from serum deprivation induced death [25, 26]. The expression of CHP2 has been reported to be significantly increased in human ovarian carcinoma cells [27] and leukemia primary cells [28] and breast cancer cells [29]. Mechanistically, overexpression of CHP2 activated AKT signaling and suppressed the transactivation of the forkhead box O3 (FOXO3/FOXO3a) transcription factor in breast cancer [29]. Besides, a study conducted 10‑gene signature including CHP2 found that high expression of CHP2 was associated with the recurrence of COAD [30]. While our results identified CHP2 was lower expressed in CRC cell lines, which was constant with the results of TCGA.

SULT sulfotransferase (SULT), a family of the enzymes catalyzing sulfonation of a variety of endogenous and exogenous substrates, comprises membrane-bound and cytosolic SULTs [31, 32]. SULT1B1 is expressed at highest levels throughout the human colon and small intestine but can also be found at moderate levels in human liver, kidney, and white blood cells [33, 34], and it is thought to be related to carcinogenesis [35]. The copy number variations of SULT1B1 significantly decreased in T4 than T1, 2 and 3 in CRC patients, and repression of SULT1B1 along with repression of UGT2B28 in CRC is thought to be related to tissue dedifferentiation [36]. As a CRC metastasis-associated gene considered in our study, we not only found that the mRNA level of SULT1B1 were decreased in CRC cell lines, but also identified its low expression was associated with a bad survival of CRC patients. Monoacylglycerol *O*-acyltransferase 2 (MOGAT2) is a member of MOGAT gene family, plays an important role in catalyzing the metabolism of triglycerides and is highly conserved in organisms. And MOGAT2 was found to have different degrees of copy number amplification and deletion in the 16 yak populations [37]. Besides, rs499974 of MOGAT2 might be relevant for the risk of type 2 diabetes through single variant analyses [38]. Moreover, MOGAT2 were identified as a differentially methylated gene in inflammatory breast cancer [39]. Chromosome 1 open reading frame 115 (C1orf115) is broadly expressed in small intestine, duodenum and 23 other tissues. C1orf115 was identified to be associated with significant changes in forced expiratory volume in 1 s (FEV1) values in chronic obstructive pulmonary disease patients for long-acting muscarinic antagonist treatment [40]. Beyond that, little is known about the roles of MOGAT2 and C1orf115 in human cancers.

## Conclusion

In summary, three modules were regarded as the most significant modules in the metastasis of CRC. Besides, CA2, CHP2, SULT1B1, MOGAT2 and C1orf115 might play important roles in the metastasis and prognosis of CRC. However, further studies are still needed to completely elucidate the biological functions of these genes and to confirm the molecular mechanisms on the development of CRC.

## Supplementary information

**Additional file 1: Figure S1.** Kaplan–Meier survival curves for 30 DEGs (ABCE1, ADAMDEC1, AURKA, C1orf115, C4orf19, CEACAM7, CLDN23, CPT2, CSTF1, DARS, DDX21, ENTPD5, ETFDH, GNL3, GPD1L, GRAMD3, HDAC2, ITM2C, LRRC19, MOGAT2, NIFK, NR3C2, PLAGL2, RBM47, RSL1D1, SLC4A4, SLC26A3, SMPDL3A, SULT1B1 and TMEM236) in greenyellow, turquoise and brown modules.

## Data Availability

The authors declare that the data supporting the findings of this study are available within the article.

## Abbreviations

CRC: Colorectal cancer
RNA-Seq: RNA-sequencing
TCGA: The Cancer Genome Atlas
WGCNA: Weighted gene co-expression network analysis
DEGs: Differentially expressed genes
DELs: Differentially expressed lncRNAs
COAD: Colon adenocarcinoma
READ: Rectal adenocarcinoma
GO: Gene ontology
KEGG: Kyoto Encyclopedia of Genes and Genomes
qRT-PCR: Quantitative real-time PCR
FC: Fold change
FDR: False discovery rate
TOM: The topological overlap measure
ME: Module eigengene
DAVID: Database for Annotation, Visualization and Integrated Discovery
GEPIA: The Gene Expression Profiling Interactive Analysis database
CA2: Carbonic anhydrase 2
CHP2: Calcineurin homologous protein isoform 2
SULT: SULT sulfotransferase
MOGAT2: Monoacylglycerol *O*-acyltransferase 2
C1orf115: Chromosome 1 open reading frame 115
FEV1: Forced expiratory volume in 1 s
