# Supplementary figures and images for: Identification of novel biomarkers affecting the metastasis of colorectal cancer through bioinformatics analysis and validation through qRT-PCR
Source: Cancer Cell Int. 2020 Mar 30;20:105. doi: 10.1186/s12935-020-01180-4 (PMC7106634; doi:10.1186/s12935-020-01180-4)

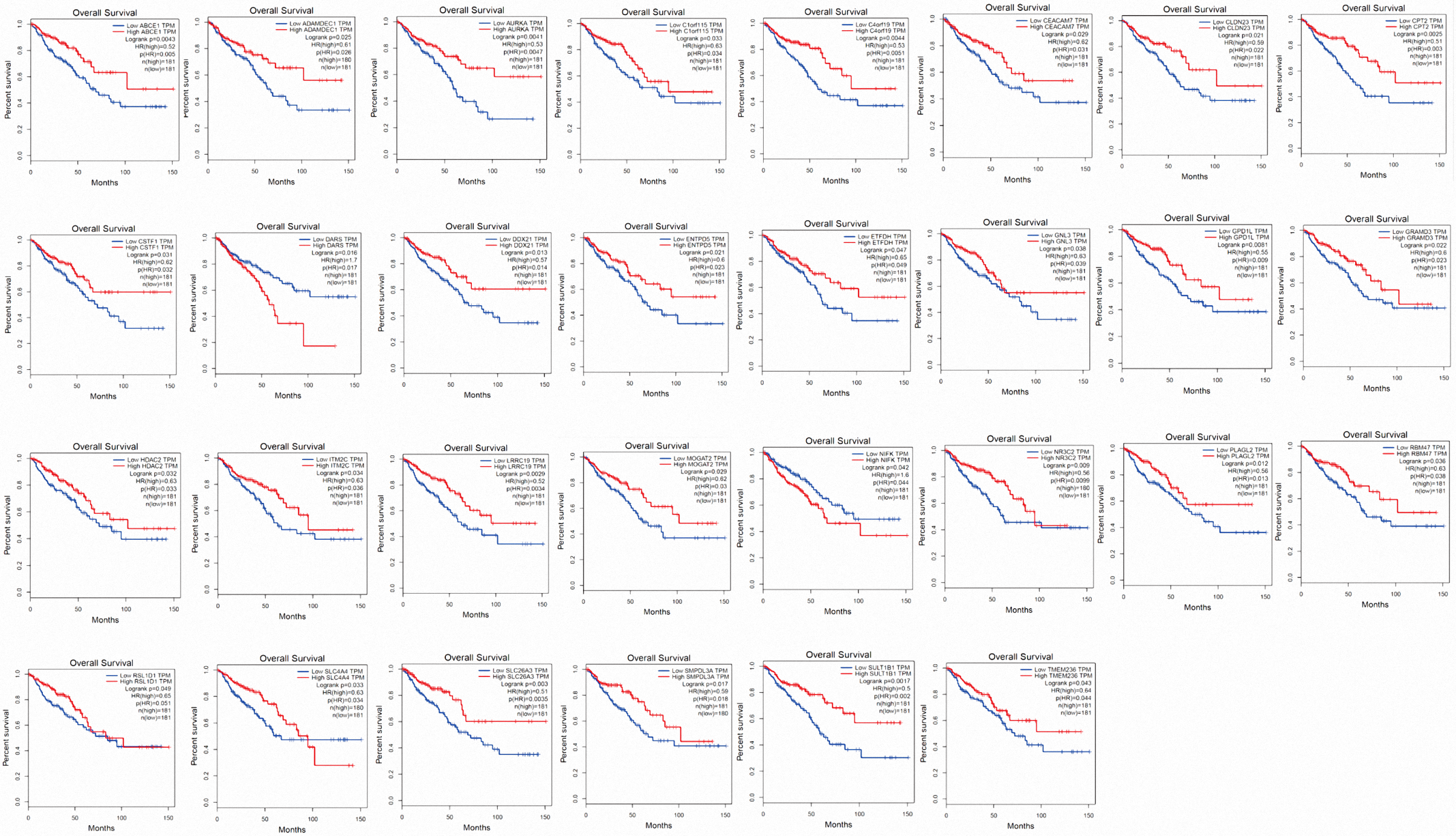

Supplement: Supplementary file 1 — Additional file 1: Figure S1. Kaplan–Meier survival curves for 30 DEGs (ABCE1, ADAMDEC1, AURKA, C1orf115, C4orf19, CEACAM7, CLDN23, CPT2, CSTF1, DARS, DDX21, ENTPD5, ETFDH, GNL3, GPD1L, GRAMD3, HDAC2, ITM2C, LRRC19, MOGAT2, NIFK, NR3C2, PLAGL2, RBM47, RSL1D1, SLC4A4, SLC26A3, SMPDL3A, SULT1B1 and TMEM236) in greenyellow, turquoise and brown modules. [file 12935_2020_1180_MOESM1_ESM.tif]
